# Traditional versus Microsphere Embolization for Hepatocellular Carcinoma: An Effectiveness Evaluation Using Data Mining

**DOI:** 10.3390/healthcare9080929

**Published:** 2021-07-23

**Authors:** Pi-Yi Chang, Chen-Yang Cheng, Jau-Shin Hon, Cheng-Ding Kuo, Chieh-Ling Yen, Jyh-Wen Chai

**Affiliations:** 1 Radiology Department, Taichung Veterans General Hospital, Taichung 40705, Taiwan; bibichang1023@gmail.com (P.-Y.C.); hubt@vghtc.gov.tw (J.-W.C.); 2 Department of Industrial Engineering & Management, National Taipei University of Technology, Taipei 10608, Taiwan; cycheng@ntut.edu.tw; 3 Department of Industrial Engineering & Enterprise Information, Tunghai University, Taichung 40704, Taiwan; tintin0986325@gmail.com; 4 Gastrointestinal Department, Taichung Veterans General Hospital, Taichung 40705, Taiwan; c9626@yahoo.com.tw

**Keywords:** liver cancer, hepatic artery embolization, embolization prognosis, data mining, decision tree, logistic regression

## Abstract

**Background**: For hepatocellular carcinoma (“HCC”), the current standard of treatment is hepatic artery embolization, generally through trans-catheter arterial chemoembolization (“TACE”). There are two types: traditional (“conventional” or “cTACE”) and microsphere (“DC bead TACE”). Unfortunately, the literature comparing the relative effectiveness of cTACE versus DC bead TACE is inconclusive, partially due to the complexity of HCC and its response to treatment. Data mining is an excellent method to extract meaning from complex data sets. **Purpose**: Through the application of data mining techniques, to compare the relative effectiveness of cTACE and DC bead TACE using a large patient database and to use said comparison to establish usable guidelines for developing treatment plans for HCC patients. **Materials and Methods**: The data of 372 HCC patients who underwent TACE in Taichung Veterans General Hospital were analyzed. The chi-square test was used to compare the difference in the effectiveness of the two therapies was compared. Logistic regression was used to calculate the odds ratios. Furthermore, using the C4.5 decision tree, the two therapies were classified into applicable fields. Chi-square test, the t-test, and logistic regression were used to verify the classification results. **Results**: In Barcelona Clinic Stages A and B cancers, cTACE was found to be 22.7% more effective than DC bead TACE. By using the decision tree C4.5 as a classifier, the effectiveness of either treatment for small tumors was 8.475 times than that for large tumors. DC bead TACE was 3.39 times more successful in treating patients with a single tumor than with multiple tumors. For patients with a single tumor, the chi-square test showed that 100–300 μm microspheres were significantly more effective than 300–500 μm. While these findings provide a reference for the selection of an appropriate TACE approach, we noted that overall accuracy was somewhat low, possibly due to the limited population. **Conclusions**: We found that data mining could be applied to develop clear guidelines for physician and researcher use in the case of complex pathologies such as HCC. However, some of our results contradicted those elsewhere in the literature, possibly due to a relatively small sample size. Significantly larger data sets with appropriate levels of granularity could produce more accurate results.

## 1. Introduction

Liver and lung cancer are the two most prevalent cancers among Asian men, and Taiwan is no exception. Cancer is the leading cause of death in Taiwan, and according to its Ministry of Health and Welfare, lung and liver cancer have always remained the top two leading types of cancers, despite falling cancer rates.

The current clinical practice for liver cancer is to treat tumors larger than 1 cm. Before initiating treatment, the degree of cirrhosis is determined based on five clinical indicators, as it is a crucial indicator for selecting the appropriate course of treatment. The Barcelona clinic liver cancer (“BCLC”) staging system is performed according to both the degree of cirrhosis (using the Child–Pugh score) and the daily physical status (using the Eastern Cooperative Oncology Group performance status scale (“ECOG PS”)), along with other indicators. A simplified and summarized BCLC staging is as follows [1]:

- Stage 0 (very early stage)
  ○ ECOG PS 0
  ○ Child-Pugh A
- Stages A, B and C (early, intermediate, and advanced, respectively)
  ○ ECOG PS 0–2
  ○ Child-Pugh A to C
- Stage D (end-stage)
  ○ ECOG PS > 2
  ○ Child-Pugh C

The hepatic artery provides 90–95% of blood and nutrition required for the survival and growth of liver cancer cells. In hepatic artery embolization, the hepatic artery is blocked to stop the blood supply to liver cancer cells; the resulting hypoxia causes shrinkage of the tumor. During embolization, chemotherapy drugs can be delivered through the catheter directly into the hepatic artery. Hepatic artery embolization is highly effective for patients with liver cancer who are not candidates for surgery, and there are lower residual levels of drugs than after conventional chemotherapy.

**Figure 1:**
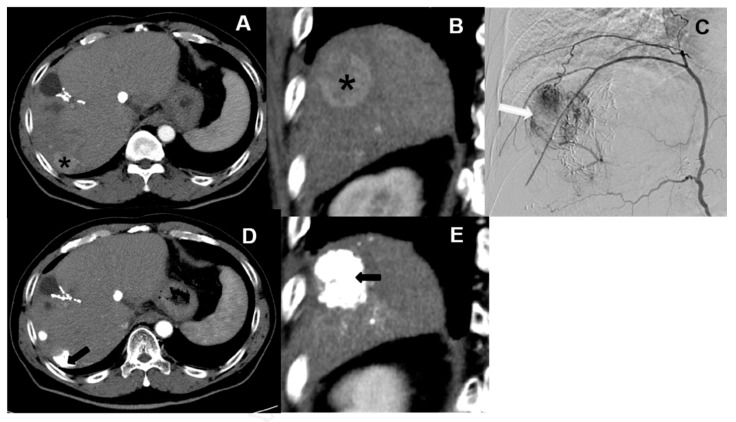
Traditional embolization (cTACE). Hepatocellular carcinomas (HCC) with arterial enhancement in segment 7 of the liver in both CT images (**A** and **B**, * star) and angiography (**C**, white arrows). TACE is performed by using conventional TACE with lipiodol accumulation (**D** and **E**, black arrows) on follow up CT.

**Figure 2:**
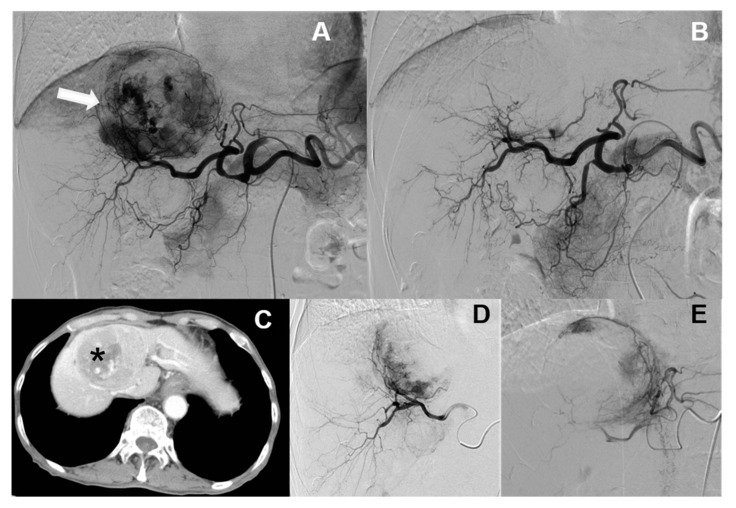
Microsphere embolization (DC bead TACE). A 7.3 cm hepatocellular carcinoma (HCC) with arterial enhancement in segment 4 and 8 of the liver in both angiography (**A**, white arrows) and CT (**C**, * star) images. TACE is performed by using drug eluting microspheres (**D** and **E**). Follow-up angiography shows complete embolization of the tumor (**B**).

Currently, hepatic artery embolization is performed using two main treatment methods, both involving trans-catheter arterial chemoembolization (“TACE”). Conventional trans-catheter arterial chemoembolization (“cTACE”) is the older of the two methods (Figure 1), whereas microsphere-loaded arterial embolization—called “DC bead TACE”, after the drug-eluting microbeads employed—is the newer (Figure 2). DC bead TACE involves the use of a microsphere-loaded drug that prolongs effective treatment times and results in better therapeutic outcomes [2]. Compared with cTACE, DC bead TACE results in better therapeutic response and delayed tumor progression; however, no significant difference has been noted in liver-related toxicity. Song et al. demonstrated the superior performance of arterial embolization performed using DC beads [3], and Ashrafi et al. indicated that DC bead TACE can result in the same tumor response as cTACE [4]. Although the latter study combined the clinical effectiveness of DC beads with that of cTACE, additional large-scale randomized controlled trials are still needed.

**Figure 3:**
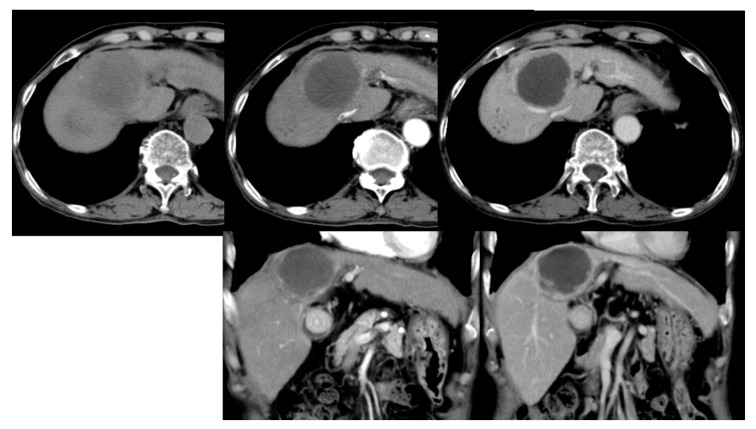
In a follow-up liver dynamic CT after treating with drug-eluting microspheres TACE, the tumor shows almost total necrosis.

According to the literature, DC bead TACE is more effective for cTACE-refractory hepatocellular carcinoma (“HCC”), particularly when the tumor is small and delayed or enhanced during angiogenesis (Figure 3). For example, Lammer et al. [5] compared the clinical efficacies of cTACE vs. DC bead TACE, both using doxorubicin, in 212 cancer patients with Child–Pugh A/B cirrhosis and large and/or multiple nodules and other inoperable cancer patients [5]. Overall, they reported that in terms of disease control, DC bead TACE was more effective than cTACE in four patients and that those adverse drug reactions were more severe after cTACE than after DC bead TACE. However, according to the chi-square analysis, the DC beads were found to have significant advantages only under certain conditions, such as a specific Child–Pugh rating and ECOG PS status, if prior curative treatments had been undergone, and in the presence of bilobar disease.

Evaluating the clinical effectiveness of chemotherapy includes a host of other variables, complicating the analysis. For instance, Muggia et al. compared the effectiveness of cisplatin-alone versus paclitaxel-alone versus combined cisplatin and paclitaxel chemotherapy in patients with late-stage ovarian cancer [6]. After patients received a 6-week treatment course, the complete and partial responses were measured every 3 weeks to determine the treatment effectiveness. Among the indicators and effects examined were neutropenia, fever, alopecia, anemia, thrombocytopenia, neurotoxicity, nephrotoxicity and gastrointestinal toxicity.

Therefore, due to the level of complexity of chemotherapy and its effects on the body, whether DC beads can completely replace cTACE in terms of curative effects remains unclear. Thus, the evaluation of which therapy is more effective under what conditions, and the identification of these conditions, can help in developing practical treatment guidelines and potential new therapies.

However, the standard practice in medical studies is to use traditional statistical analysis to determine drug effectiveness, death rates, etc. While traditional statistical analysis is adequate for finding relationships between variables and how significant those relationships are, it is not particularly well-suited for modeling complex systems in a way that is predictive in a practical sense. Rather, this is the purview of data mining and data exploration.

Data mining and exploration are a series of processes to explore the added value of information from a database by extracting and recognizing what is important or interesting in ways that cannot be known by traditional means. Data mining is also commonly known as “knowledge discovery in databases” (“KDD”) and is an important tool for manipulating data to extract important information according to the user’s purpose.

Data mining has become especially popular in recent years because of its ability to convert large amounts of data into some useful information and knowledge. This has been particularly useful in scientific research that employs large databases. The main difference between data mining/exploration and traditional statistics is the amount of data processed, with the former being very well-suited towards large databases. Data mining and exploration can create powerful predictive models of complex systems with large data sets.

The application of data mining to HCC is relatively new. A PubMed search of the terms “HCC”, “liver cancer” and “data mining” yielded less than 200 results at the time of writing. The vast majority of the literature on this matter is concerned with the application of data mining to gene expression and regulation [7,8], biomarkers [9,10] and predictors [11,12], but relatively little work has been done on the effectiveness of HCC treatments. There are a few studies regarding medications such as lenvatinib [13] and sorafenib [14], but the data-mining studies on the effectiveness of other modes of treatment are still rare.

Prior studies have shown that DC bead TACE has advantages over cTACE only under certain conditions, but due to the complexity of chemotherapy studies, clear guidelines advising the use of one over the other and under what circumstances are still lacking. This is likely due to the complexity of both HCC and its response to different modes of treatment, both of which may remain relatively opaque to traditional statistical methods. This study aims to use clinical indicators and data exploration to re-examine the effectiveness of cTACE and DC bead TACE and verify the current clinical data on liver cancer arterial embolization. This study refers to the literature on cancer prognosis [15] and employs decision trees, neural networks and logistic regressions to predict and compare data.

## 2. Methods and Materials

The present work is a retrospective study and was approved by the Institutional Review Board of Taichung Veterans General Hospital (IRB No. CE17306A), waiving the requirement for informed consent. Patient data were collected from the 2010–2017 Informatics Research and Development Center of Taichung Veterans General Hospital. We collected the data of liver cancer patients who underwent hepatic artery embolization performed using DC Bead TACE/yttrium 90 microsphere carrier drug-carrying therapy (the “new” therapy) and cTACE oil–water carrier drug therapy (the “old” therapy), as well as those of patients who underwent both the therapies. AIDS patients with severely poor prognoses were excluded from this study.

After exclusion, we collected the data of 372 patients, and defined the null hypothesis (H0) as “microsphere embolization therapy is more effective than traditional therapy” and the alternative hypothesis (H1) as “traditional therapy is more effective than microsphere embolization therapy”. The Attributes or Input items of this study were liver cancer staging (unit: period), tumor size (unit: cm), tumor number (unit: units) [16], new therapy/old therapy [17], microsphere size (unit: μm) [4] and hepatitis type. The Output items were the prognostic indicators of liver cancer [15] and the effects of cancer treatment.

In the first step of logistic regression, the regression coefficient (β1, 2, 3…) was calculated from the training data; then, the probability of verification data being imported into the model with the coefficient was predicted. The predicted odds were then calculated. In medical diagnostic data mining, when the C4.5 decision tree is compared with CART (Classification and Regression Trees), although they both exhibit a similar classification accuracy, the C4.5 algorithm performs better in controlling the scale of the decision tree and generates rules that are more understandable. As the object of this study was the application of medical diagnostic data mining towards evaluating the effectiveness of two different cancer therapies, the C4.5 (J48) decision tree using the ID3 system was selected.

### 2.1. ID3 Algorithm

The core concept behind the ID3 algorithm is, “the greater the information entropy, the murkier the data”. The following definitions were used:

Original information entropy:

(1) $$\;I(p,\;n)=-((\frac{p}{n+p})\;x\;log_{2}\left(\frac{p}{n+p}\right)+\;(\frac{n}{n+p})\;x\;log_{2}\left(\frac{n}{n+p}\right))$$

Expected post processing information entropy:

(2) $$E(x)=\sum_{i=1}^{n}(\left(\frac{n_{i}+p_{i}}{n+p})\;x\;I\left(n_{i},p_{i}\right)\right)$$

Data gain:

(3) Gain(x)=I(p, n)−E(x)

The data gain of each attribute is the decision parameter of the decision tree branch; that is, the maximum gain of each attribute is the branch node, indicating that the attribute can be used to minimize the turbidity of the data. The gain of all the attributes was calculated and compared.

### 2.2. C4.5 (J48) Algorithm

ID3 has a partiality problem, such as the ID number. If an ID is used for each datum as the branch point, then the gain will be maximized. Another instance is that if the self-variation is the same in the data, then the gain will be the minimized. To prevent this issue, C4.5 changes the gain ratio to makes branch decisions. The amount of gain/self-variation of the information entropy itself (i.e., considering the problem of self-variation of the body quality) can prevent the occurrence of decision paralysis.

The core concept used herein: while adhering to the core concept of ID3, consider the information entropy and eliminate the problem of decision paralysis.

Self-variation information entropy:

(4) $$\mathrm{SI}(X)=-\sum_{j=1}^{v}(\left(\frac{N_{j}}{N})\;xlog_{2}\left(\frac{N_{j}}{N}\right)\right)$$

Information gain rate:

(5) $$\mathrm{GR}\left(X\right)=\frac{\mathrm{Gain}(X)}{\;\mathrm{SI}(X)}$$

The data gain rate of each attribute is the decision parameter of the decision tree branch; that is, the maximum gain rate of each attribute is the branch node, indicating that the attribute can be divided to clarify the data as soon as possible. Finally, 10-fold cross validation is the method used to divide training and verification data. The area under the confusion matrix, the area under the receiver operating characteristic curve (“ROC curve”), and the area under the precision-recall curve (“PR curve”) [18] were used and compared for the three models in this study.

From Table 1, we can understand the operation model of the overall confusion matrix and then the judgment indicators extended by the confusion matrix. We defined each indicator according to the code used in the table as follows:

First, from the prediction accuracy surface (C, D surface):

(6) $$\mathrm{Precision}=\frac{TP}{C}\;=\frac{TP}{TP+FP}$$

(i.e., to predict the correct middle).

(7) $$\mathrm{Recall}=\frac{TN}{D}\;=\frac{TN}{TP+FN}$$

(i.e., the prediction error is in the middle).

Introduced from the real side (A, B side):

(8) $$\mathrm{True}\;\mathrm{positive}\;\mathrm{rate}\;(\mathrm{TPR})=\frac{TP}{A}\;=\frac{TP}{TP+FN}$$

False negative rate:

(9) $$(\mathrm{FNR})=\frac{\mathrm{FN}}{A}\;=\frac{\mathrm{FN}}{TP+FN}$$

True negative rate:

(10) $$(\mathrm{TNR})=\frac{TN}{B}\;=\frac{TN}{FP+TN}$$

False positive rate:

(11) $$(\mathrm{FPR})=\frac{FP}{B}\;=\frac{FP}{FP+TN}$$

The overall model accuracy (E-side):

(12) $$\mathrm{Accuracy}\;\mathrm{rate}=\frac{TP+TN}{E}=\frac{TP+TN}{TP+FN+TN+FP}$$

Precision is data taken based on a lack of information. In binary classifications, precision can be made equal to positive predictive values. Recall is deletion data that were successfully retrieved from data relevant to the query. In binary classification, recall is known as “sensitivity”. The appearance of relevant data taken agrees with the query that can be seen with recall. Accuracy rate is a percentage of the total data identified and assessed. The likelihood ratio (“LR”) derived from the TPR (the amount of positive data correctly classified by the system), FNR (the amount of negative data but classified incorrectly by the system), TNR (the amount of negative data correctly classified by the system), and FPR (the amount of positive data but classified incorrectly by the system) can be judged based on data calculated from the LR value.

LR (+) = TPR/FPR = TP/(TP + FN)/FP/(FP + TN)(13)

LR (−) = FNR/TNR = FN/(TP + FN)/TN/(FP + TN)(14)

The areas under the ROC and PR curves are crucial for judging the accuracy of the model. If the area is 0.5, the probability of guessing is equal, and if the area is below 0.5, then the probability of guessing is lower. The main difference between the two curves is the sensitivity of positive and negative samples. For example, when the positive to negative sample ratio is increased, the ROC curve does not change significantly, while the PR curve changes severely. Therefore, when differences between positive and negative samples are large, the PR curve is more suitable.

## 3. Results

### 3.1. Baseline Comparison of cTACE and DC Bead TACE

Analyses were first performed on the complete data set without any further exclusions. As shown in Table 2, the proportions of “old” and “new” treatments (cTACE and DC bead TACE, respectively) were compared with their chi-square distribution. The “cTACE is more effective” hypothesis was valid 68.2% of the time (for brevity, “effective ratio”) and was invalid 31.8% of the time (for brevity, “invalid ratio”). The DC bead TACE effective ratio was 45.6% and the invalid ratio was 54.54%. According to the two-tailed test, cTACE was significantly better than DC bead TACE (*p* < 0.05).

To evaluate the impact of BCLC cancer staging on effectiveness, the data of patients with either BCLC stage C or D cancer were excluded; then, the effectiveness of the two treatments was compared. The number of patients who underwent cTACE did not change, indicating that patients with either stage C or D cancer did not undergo the “old” embolization therapy. Thus, the cTACE effective ratio did not change. However, the number of patients who underwent the new therapy changed from 171 to 143, a difference of 28 patients with stage C or D cancer. However, the DC bead TACE effective ratio only changed from 45.6% to 45.5%. Thus, no significant differences were observed in the effectiveness of old and new therapies for patients with stage C or D cancer. The effective ratios of patient pools with stage C or D cancer were similar to those without, indicating a significant advantage of cTACE for patients with stage A or B cancer. The results of the logistic regression are shown in Table 3, which shows the regression coefficients for each group.

### 3.2. cTACE Limitations and Applicable Segments

The C4.5 decision tree and real medical records were used to determine whether tumor size affected the effectiveness of either treatment on HCC. Tumors were considered “small” if they were 9.3 cm or smaller and were “large” otherwise. We began by examining the effect of tumor size using the statistical mean *t* test. The average tumor size that showed an effective curative effect was compared with that which did not show one, and chi-square test results are shown in Table 4. Therefore, the results implied that if cancer is graded using the BCLC staging system, those with stages A and B cancer can undergo cTACE if the tumor size is 9.3 cm or smaller and that other treatments should be recommended if the tumor size is larger than 9.3 cm.

Logistic regression was used to compare the difference in effectiveness between the two treatments. The odds ratios of the logistic regression output indicated that the rate of effectiveness for small tumors was 8.475 times better than the large tumors. Therefore, if the tumor size is 9.3 cm or smaller in patients with stage A or B cancer, then they should undergo cTACE. If the tumor is larger than 9.3 cm, then the success rate of cTACE drops to 11.8%. This result could provide a reference or guideline for oncologists to choose the most appropriate therapy.

The effectiveness of DC bead TACE (aka “microsphere therapy”) was determined using a C4.5 decision tree. The model was divided into two layers. The first node was divided by the number of tumors, and the second node was divided by the size of the ball. A preliminary analysis showed that the decision tree could find that different sizes of microspheres affected the effectiveness of treatment. However, the accuracy of the model was 59.1%, the false positive rate was 0.41, the accuracy was 0.594, and the recall rate was 0.591. The prediction ability of the area under the ROC curve and the area under the PR curve surface were 0.6 and 0.438, respectively.

**Figure 4:**
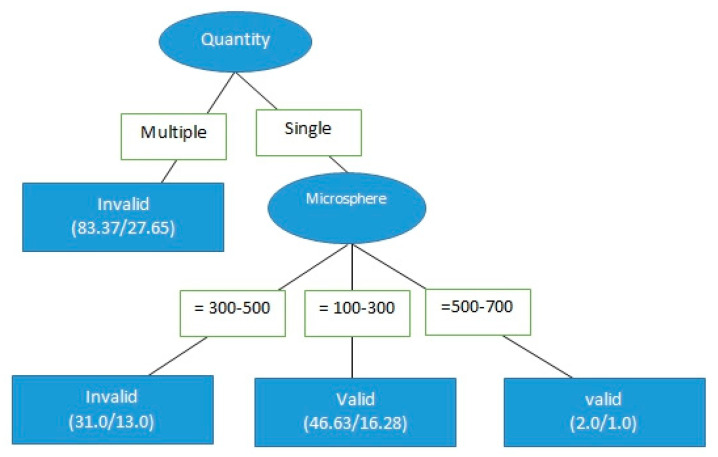
Decision tree classification of the effectiveness of DC bead TACE with single-sized microspheres on single tumors.

Because the accuracy of the first iteration was so low, the mixed-sized microsphere cases were then excluded from the next iteration. This was done because there were very few cases using mixed-size microspheres. The accuracy of the model improved from 59.1% to 65%. Compared with the model tree in mixed-sized microspheres, the scale of the model tree was substantially decreased. The applicable conditions of the model are shown in Figure 4.

**Figure 5:**
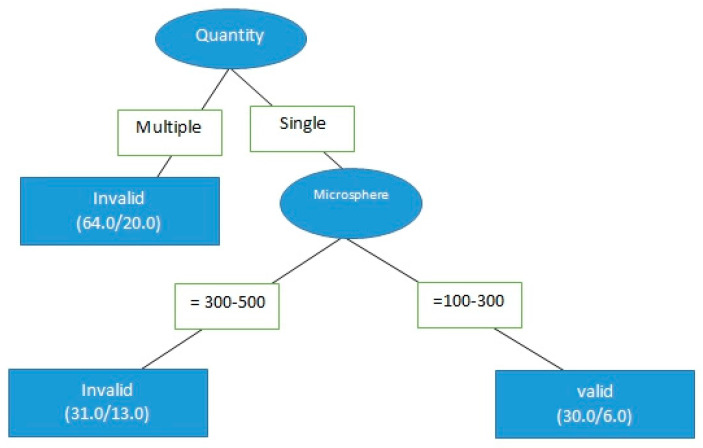
Decision tree classification, the same as the previous iteration but with 500–700 μm-sized microspheres also excluded.

To further optimize the model, the data were examined for further possible small but potentially significant exclusions. Microspheres of 500–700 μm in size were used only in 5 patients out of the original 130 who received DC bead TACE, and so they were excluded as well. The classification results are shown in Figure 5. The accuracy of the model increased from 65% to 70.4%, and those of other overall indicators also increased sharply. These findings indicated that the amount of attribute data alone did not affect the accuracy of the model.

With these exclusions in place, the chi-square test was used to examine the differences in the effectiveness of tumor treatment, with the degree of difference determined using logistic regression. Then the chi-square test and logistic regression were performed for microsphere sizing according to the results of the decision tree classification, in order to provide a more complete result for medical reference. The results of the chi-square test for DC bead TACE treatment are shown in Table 5. The single-tumor treatment invalid ratio was 39.3%, whereas the effective ratio was 60.7%. The invalid ratio of multiple tumors was 68.8%, whereas the effective ratio was 31.3%, and there was a significant difference between the single-tumor and multiple-tumor (chi-square test = 0.01).

## 4. Discussion

### 4.1. Baseline Comparison of cTACE and DC Bead TACE

In terms of cTACE and *DC* bead *TACE* comparison, this result differed from those of previous studies. In the present study, the difference between the validity of the two hypotheses was 22.7% in favor of the “cTACE is more effective” hypothesis. This statistical result shows that the old cTACE method has a therapeutic advantage, but with the caveat of it only occurring when the stage of cancer is not considered.

To evaluate the impact of BCLC cancer staging on two treatments, the regression coefficients of the traditional therapy showed that it is 1.0669 times more effective than that of the microsphere therapy. The regression coefficients of DC bead TACE mixed-sized microsphere groups of 100–300 μm + 300–500 μm and 100–300 μm + 500–700 μm in size were relatively high. Data on patients with multiple tumors and their sizes and locations were not available. In both cases, there is a high probability of producing effective results with more adequate clinical data. In the future, it may be possible to show that the number of tumors (as opposed to single vs. multiple) is a more influential factor in the effectiveness of both treatments and to explore the effectiveness of mixed-sized microspheres in DC bead TACE. These results indicate two possibilities. First, patients with stage A or B cancer who undergo cTACE have a high probability of controlling their cancer, and patients with stage C or D cancer do not undergo cTACE. Therefore, we could not compare the advantages and disadvantages of the old and new treatments in patients with stage C or D cancer and suggest this be an area of future research. The second possibility is that although microsphere therapy provides the benefit of expanding the field of liver cancer embolization, its treatment effect could be relatively low. The results above show an effectiveness rate of only 45.6% overall and administering this therapy to cancer patients with congenital conditions is difficult. Even after excluding cancer patients with congenital conditions who are difficult to treat, and patients with stage C or D disease, microsphere therapy still showed a low relatively low therapeutic effect, which could also be a topic for further study.

### 4.2. cTACE Limitations and Applicable Segments

The C4.5 decision tree and real medical records were used to determine whether tumor size affected the effectiveness of either treatment on HCC. These results indicated that microsphere therapy has significant advantages in the treatment of single-tumor patients. More research into the risks and effectiveness of DC bead TACE in multiple-tumor patients is necessary, particularly in terms of the question of whether the number of tumors impacts effectiveness.

Logistic regression was then used to calculate the odds ratio of the number of tumors affecting treatment effectiveness. Treating a single tumor was 3.39 times more successful than treating multiple tumors. However, we recognized that this odds ratio was calculated from a fairly limited data set. Although the accuracy of the model was approximately 70.4%, the prediction ability was not very satisfactory. Having more clinical data in the future is necessary to improve model prediction.

Next, only the data of the 61 patients who were treated for single-tumor HCC with DC bead TACE using either 100–300 μm or 300–500 μm microspheres were analyzed to determine the effect of microsphere size on treatment effectiveness. The data were divided into two different microsphere sizes (30 records for 100–300 μm and 31 records for 300–500 μm, as per Figure 2), for a total of 61 records. The result of the chi-square test is shown in Table 6. For microspheres that were 100–300 μm in size, the invalid ratio was 20% and the effective ratio was 80%. For microspheres that were 300–500 μm in size, the invalid ratio was 58.1%, whereas the effective ratio was 41.9%; the chi-square test result was 0.02, indicating a significant difference, that is, microspheres sized 100–300 μm were more therapeutically effective than those sized 300–500 μm in size for a single tumor. The extent of the differences in effectiveness should be further explored.

The odds ratios of the microspheres that were 100–300 and 300–500 μm in size were calculated using a logistic regression. The success rate of treatment with DC bead TACE using 100–300 μm microspheres was 3.412 times of that using 300–500 μm microspheres. The accuracy of this model was approximately 64.8%; however, its prediction ability still needs to be strengthened.

One limitation of this study was the relatively small sample size. Although the factors surrounding HCC and TACE are varied and complex, the strengths of data mining lie also in its ability to analyze large data sets. While the initial population was reasonably large (372), by the time the analysis had reached the point of parsing different aspects of microsphere therapy, the populations had shrunk considerably. In the future, larger populations to which data mining can be applied would probably yield more interesting results.

## 5. Conclusions

This study found that the effective rate of the traditional cTACE therapy is 1.0669 times better than that of the DC bead TACE microsphere therapy. The effectiveness of either treatment is also affected by tumor size; tumors 9.3 cm or smaller responded much better treatment than those that were larger. Furthermore, microsphere therapy was found to be 2.8719 times more effective in treating single tumors than multiple tumors. Microspheres that were 100–300 and 300–500 μm in size had a high effective probability, and their effectiveness should be verified in future studies. Therefore, for patients with stage A or B HCC, the use of cTACE is supported by our data, showing a significant advantage of 22.7%. The data also support the treatment paradigm that patients with stage C or D HCC respond better to DC bead TACE, with the caveat that having multiple tumors has a negative impact on effectiveness. The data also indicate that microspheres larger than 500 μm are not as effective as smaller microspheres. As a result, we conclude that data mining techniques can be effectively used to evaluate treatment effectiveness and create clear clinical guidelines for physicians. However, larger data sets are recommended for better accuracy.

## Figures and Tables

**Table 1:** Confusion Matrix.

		Prediction		
		Correct	Error	
Real	Correct	True Positive (TP)	False Negative (FN)	TP + FN = A
	Error	False Positive (FP)	True Negative (TN)	FP + TN = B
		TP + FP = C	FN + TN = D	A + B = C + D = E

**Table 2:** cTACE vs DC Bead TACE Treatments.

						cTACE vs. DC Bead Crosstab	
						cTACE	DC Bead
Valid/Invalid	Invalid	Number				64	78
		Within the cTACE and DC bead treatments				31.80%	54.54%
		Overall percentage				45.10%	54.90%
	Valid	Number				137	65
		Within cTACE vs. DC bead treatments				68.20%	45.60%
		Overall percentage				67.80%	32.20%
**Chi-Square Test**							
				**Degrees of**	**Asymptotic**	**Precise**	**Precise**
			**Value**	**Freedom**	**Significant**	**Significant**	**Significant**
					(two-tail)	(two-tail)	(single-tail)
Pearson chi-square			17.770 ^a^	1	<0.001		
Continuity correction ^b^			16.845	1	<0.001		
Approximate ratio			17.793	1	<0.001		
Fisher’s accurate verification						<0.001	<0.001
Linear connection			17.718	1	<0.001		
The valid observation number			344				

^a^ The expected number of 0 grid (0%) was less than 5, and the minimum expected number was 59.03. ^b^ could only calculate 2 × 2 forms.

**Table 3:** Logistic regression output results (odds ratios expressed as regression coefficients).

Variable	Class Valid
Hepatitis = B	0.8758
Hepatitis = C	1.1042
Hepatitis = non-B non-C	0.9071
Hepatitis = B + C	1.3726
BCLC stage = B	0.9389
BCLC stage = C	1.2431
BCLC stage = A	0.9672
BCLC stage = D	1.7284
Quantity = single	2.8719
Size	0.9137
Microsphere = 300–500	0.2855
Microsphere = 300–500 + 500–700	1.5049
Microsphere = 100–300	0.5364
Microsphere = 500–700	0.4341
Microsphere = 100–300 + 500–700	9.99 × 10^19^
Microsphere = 100–300 + 300–500	46,749,492.226
Microsphere = 0	1.0669
New vs. old treatments	1.0669

**Table 4:** T-test result, Tumor Size vs. Effectiveness.

					Tumor Size vs. Effectiveness Crosstab		
					**≤9.3 cm**		**>9.3 cm**
	Invalid	Number			51		13
		Within size tumor 9.3			27.70%		76.50%
Valid/		Overall percentage			79.70%		20.30%
Invalid	Valid	Number			133		4
		Within size tumor 9.3			72.30%		23.50%
		Overall percentage			97.10%		2.90%
**Chi-Squared Test**							
				**Degrees of**	**Asymptotic**	**Precise**	**Precise**
			**Value**	**Freedom**	**Significant**	**Significant**	**Significant**
					(two-tail)	(two-tail)	(single-tail)
Pearson chi-square			17.044 ^a^	1	<0.001		
Continuity correction ^b^			14.871	1	<0.001		
Approximate ratio			15.749	1	<0.001		
Fisher’s accurate verification						<0.001	<0.001
Linear connection			16.959	1	<0.001		
The valid observation number			201				

^a^ The expected number of 0 grid (0%) is less than 5 and the minimum expected number is 5.41. ^b^ could only calculate 2 × 2 forms.

**Table 5:** Microsphere treatment (DC bead TACE), excluding mixed-sized and 500–700 μm-sized microspheres.

			Tumor Number Crosstab			
			Single		Multiple	
	Invalid	Number	24		44	
		Within number of tumors	39.30%		68.80%	
Valid/		Overall percentage	35.30%		64.70%	
Invalid	Valid	Number	37		20	
		Within number of tumors	60.70%		31.30%	
		Overall percentage	64.90%		35.10%	
**Chi-Square Test**						
			**Degrees of**	**Asymptotic**	**Precise**	**Precise**
		**Value**	**Freedom**	**Significant**	**Significant**	**Significant**
				(two-tail)	(two-tail)	(single-tail)
Pearson chi-square		10.887 ^a^	1	0.001		
Continuity correction ^b^		9.734	1	0.002		
Approximate ratio		11.046	1	0.001		
Fisher’s accurate verification					0.001	0.001
Linear connection		10.8	1	0.001		
The valid observation number		125				

^a^ The expected number of 0 grid (0%) was less than 5 and the minimum expected number is 27.82. ^b^ can only calculate 2 × 2 forms.

**Table 6:** Microsphere therapy, single-tumor only, mixed-size and 500–700 μm-sized microspheres excluded.

					Effectiveness Crosstab	
					Invalid	Effective
		Number			6	24
	100–300 μm	Within the size of microsphere			25.00%	64.90%
microsphere		Overall percentage			20.00%	80.00%
size		Number			18	13
	300–500 μm	Within the size of microsphere			75.00%	35.10%
		Overall percentage			58.10%	41.90%
**Chi-Square Test**						
			**Degree of**	**Asymptotic**	**Precise**	**Precise**
		**Value**	**Freedom**	**Significant**	**Significant**	**Significant**
				(two-tail)	(two-tail)	(single-tail)
Pearson chi-square		9.256 ^a^	1	0.002		
Continuity correction ^b^		7.73	1	0.005		
Approximate ratio		9.583	1	0.002		
Fisher’s accurate verification					0.004	0.002
Linear connection		3.105	1	0.003		
The valid observation number		61				

^a^ The expected number of 0 grid(0%) is less than 5 and the minimum expected number is 11.8. ^b^ can only calculate 2 × 2 forms.
